# A Ferroptosis Molecular Subtype-Related Signature for Predicting Prognosis and Response to Chemotherapy in Patients with Chronic Lymphocytic Leukemia

**DOI:** 10.1155/2022/5646275

**Published:** 2022-07-06

**Authors:** Han Gong, Heng Li, Qin Yang, Guangxiong Zhang, Hong Liu, Zekang Ma, Hongling Peng, Ling Nie, Xiaojuan Xiao, Jing Liu

**Affiliations:** ^1^Department of Hematology, The Second Xiangya Hospital; Molecular Biology Research Center, Center for Medical Genetics, School of Life Sciences; Hunan Province Key Laboratory of Basic and Applied Hematology, Central South University, Changsha 410011, China; ^2^School of Medical Laboratory, Shao Yang University, Hunan Province 422000, China; ^3^Department of Dermatology, Hunan Key Laboratory of Skin Cancer and Psoriasis, Hunan Engineering Research Center of Skin Health and Disease, Xiangya Clinical Research Center for Cancer Immunotherapy, Xiangya Hospital, Central South University, Changsha, Hunan 410008, China; ^4^Department of Hematology, Xiangya Hospital, Central South University, Changsha, Hunan 410008, China

## Abstract

Ferroptosis is a type of regulated cell death catalyzed by the iron-dependent accumulation of lipid hydroperoxides to lethal levels. Chronic lymphocytic leukemia (CLL) is a chronic lymphoproliferative disorder. However, the understanding of ferroptosis in CLL remains largely poor. In this study, we investigated the stratification and prognostic role of ferroptosis-related genes in CLL patients of ICGC cohort. We obtained fourteen genes with prognostic value by screening 110 ferroptosis-related genes (FRGs). Based on the expression profiles of these 14 genes, we classified CLL patients into two clusters. Most of the FRGs were highly expressed in cluster 1, and cluster 1 was associated with better overall survival (OS). Subsequently, we developed an eight-gene signature (TP63, STEAP3, NQO1, ELAVL1, PRKAA1, HELLS, FANCD2, and CDKN2A) by using LASSO analysis. This risk signature divided CLL patients into high- and low-risk groups. We used Cox regression analysis and ROC analysis demonstrated the risk signature was reliable and robust. And we validated the risk model in an external cohort (GSE22762). We also conducted enrichment analysis and genomic mutation analysis. Finally, we explored the potential effect of chemotherapy between the two risk groups. Our study contributed to understanding the role of ferroptosis in CLL and facilitated personalized and precision treatment.

## 1. Introduction

Ferroptosis is a new type of cell death catalyzed by the iron-dependent accumulation of lipid hydroperoxides to lethal levels, which was firstly defined in 2012 [1, 2]. Many studies have provided evidence that ferroptosis represents an option to eliminate leukemic cells but not normal hematopoietic cells because leukemic cells demand a higher level of iron [3]. For example, diffuse large B-cell lymphoma cells [4] and acute myeloid leukemia cells [5] and menin-mixed-lineage leukemia [6] are sensitive to ferroptosis induced in hematologic malignancies. However, the susceptibility to ferroptosis is remarkably different in various cancer cells [7] and the knowledge of the lymphocytic leukemia (CLL) susceptibility to ferroptosis is still lacking.

Lymphocytic leukemia (CLL) is a lymphoproliferative malignancy, characterized by the presence of clonal CD5+CD23+ B lymphocytes in the peripheral blood [8]. In western countries, CLL is the commonest leukemia with over 15000 newly diagnosed cases and about 4500 deaths in 2020 [9]. Despite great progress having been made in recent years, CLL remains an incurable disease [10]. One-third of patients with indolent CLL probably do not need medical attention, another one-third may progress in many years after initial diagnosis, and the last one-third need immediate medical attention. Therefore, risk stratification of CLL patients to guide clinical follow-up options, either to treat or to wait and watch, is extremely important [11]. The highly variable clinical course of the disease has made difficulty to predict survival for patients with CLL. Currently, two clinical staging systems, Rai and Binet, have been proved to be good predictors to stratify patients with a good correlation in terms of survival time; however, due to the recent progress in CLL therapy, these staging systems are becoming insufficient [9]. In recent years, biological markers, genetic markers, and comprehensive prognostic scores (for instance, CLL International Prognostic Index (CLL-IPI)) have been applied to predict response to treatment and survival time. However, those prognostic tools are still imperfect. To determine the best treatment strategy for individual patient, there is still a critical need for prognostic models that better stratify patients according to the likely outcome [12].

Recently, some studies have revealed the prognostic value of ferroptosis-related genes in cancers such as hepatocellular carcinoma [13], glioma [14], and esophageal adenocarcinoma [15], but not in CLL. In the current study, we analyzed the ferroptosis-related genes and corresponding clinical data in CLL based on International Cancer Genome Consortium (ICGC) and GSE22762. Our study indicated that ferroptosis-related genes can be used to stratify CLL patients based on overall survival (OS). Then, we developed a risk signature containing eight ferroptosis-related genes for predicting the OS of CLL patients. At last, we systematically compared the differences (including biology function, immunity, mutation status, and drug susceptibility) between high- and low-risk CLL patients that were stratified by the ferroptosis-related eight-gene signature.

## 2. Material and Methods

### 2.1. Data Collection

The transcriptome profiles, mutation data, and corresponding clinical data of the CLL patients were acquired from the CCLE-ES dataset of ICGC database (https://dcc.icgc.org/) and the GEO database of NCBI (https://www.ncbi.nlm.nih.gov/). Ferroptosis-related genes were obtained from FerrDb (http://www.zhounan.org/ferrdb/) and related literature [1, 2].

### 2.2. Identification of Ferroptosis-Related Prognostic Genes and Patient Subgroups

When performing the R package “survival” analysis, the median value was used as the cutoff value to calculate the relationship of all FRGs with prognosis. A Kaplan-Meier curve was used to identify the prognostic value of FRGs and we obtained 14 genes with *p* < 0.05. Fourteen ferroptosis-related genes with significant prognostic value were selected to classify patients. R package “ConsensusClusterPlus” was used to cluster patients of ICGC cohort and then PCA method was utilized to verify the subgroups.

### 2.3. Construction and Validation of a Ferroptosis-Related Risk Model

The least absolute shrinkage and selection operator (LASSO) method and R package “glmnet” were ducted to screen key prognostic genes based on the fourteen ferroptosis-related genes. After adjusted the penalty parameter via ten-fold cross-validation to narrow the number of genes, a risk model containing eight ferroptosis-related prognostic genes was established based on the best lambda value. The following formula was used to calculate the risk score of patients in the ICGC and GSE22762 cohort: Risk score = Coef_gene1_ × Exp_gene1_ + Coef_gene2_ × Exp_gene2_ + ⋯+Coef_gene8_ × Exp_gene8_. Coef was the coefficient with the LASSO method and Exp was the gene expression value. Subsequently, ROC and univariate and multivariate Cox regression were used to analyze and verify our risk model.

### 2.4. Functional Enrichment Analysis

The eight ferroptosis-related prognostic genes classified CLL patients into high- and low-risk groups. R package “DESeq2” was performed to identify DEGs (FDR <0.05 and |log_2_FC| >1) between the two groups. Next, the R package “clusterProfiler” was used to analyze DEGs with gene ontology (GO) and Kyoto Encyclopedia of Genes and Genomes (KEGG) pathways. The gene set “50 hallmark gene sets” downloaded from msigdb (http://www.gsea-msigdb.org/) was used to perform GSEA analysis.

### 2.5. Estimation of Tumor Immune Microenvironment (TIM) Scores and Immune Cell Fractions

R package “ESTIMATE” was performed to calculate TIM scores for CLL patients. CIBERSORT algorithm was conducted to calculate the proportions of 22 immune cell types (Supplementary Table S1) in the ICGC cohort. Moreover, Spearman's analysis was utilized to estimate the correlation between the risk score and TIM scores or immune cell fractions.

### 2.6. Statistical Analysis

R package “survival” and “survminer” were used to perform the Kaplan-Meier survival analysis in CLL patients and the *p*-value was based on the log-rank test. The R package “survivalROC” was utilized to calculate the area under the curve (AUC) value for 1-, 3-, and 5-year survival. Student's *t*-test was used to compare differences between different patient groups. R v4.0.3 was used for all analyses and *p* < 0.05 was considered statistical significance for all tests.

## 3. Results

### 3.1. CLL Classification Based on the Differential Expression of Ferroptosis-Related Genes

**Figure 1:**
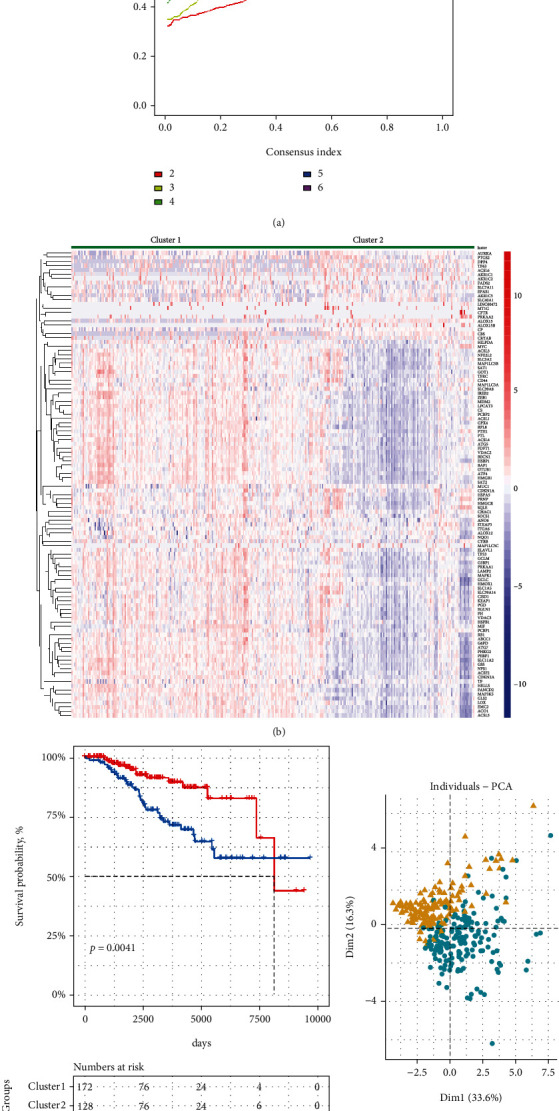
Identification of consensus clusters by ferroptosis-related genes. (a) The consensus curve plot showed the consensus clustering cumulative distribution function for *k* =2–9. (b) The heat map showed the gene expression patterns of all FRGs. (c) Survival analysis of CLL patients in clusters 1 and 2 in ICGC cohort. (d) PCA analysis for the two clustering patients. The cluster 1 was clearly separated from the cluster 2.

To explore the prognostic value of FRGs in patients with CLL, the ICGC database including 300 CLL patients was used as a training cohort. Fourteen prognostic genes (*p* < 0.05) were obtained from 110 ferroptosis-related genes using R package “survival”. Then, R package “ConsensusClusterPlus” was performed to conduct the consensus clustering analysis based on the fourteen prognostic genes. Two clusters were identified because the CDF value was smaller when *k* was equal to 2 (Figure 1(a)). We compared the expression pattern of all ferroptosis-related genes in the two clusters, and most of the FRGs were highly expressed in cluster 1 (Figure 1(b)). The Kaplan-Meier curve showed that the CLL patients of cluster 2 had a poorer OS rate than the CLL patients of cluster 1 (Figure 1(c)). To further demonstrate our classification strategy, PCA method was conducted and we could observe a clear separation between the two clusters of patients (Figure 1(d)).

### 3.2. Construction of a Ferroptosis-Related Eight-Gene Signature in the ICGC Cohort

**Figure 2:**
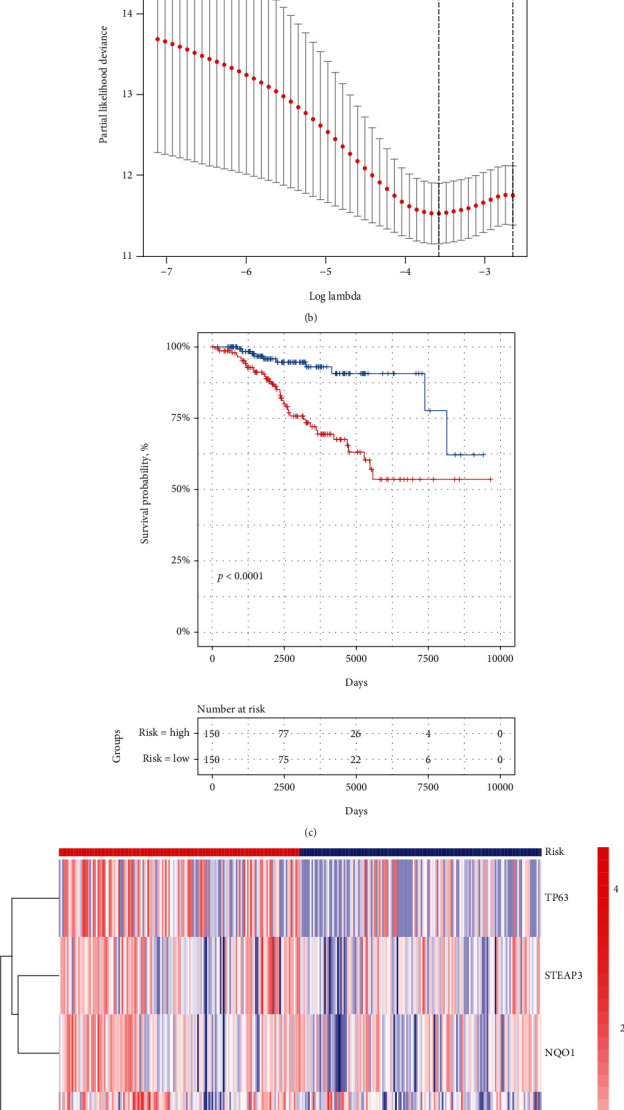
Risk signature of eight ferroptosis-related genes. (a) Coefficients calculated by the LASSO analysis. (b) Coefficient spectrum of 8 genes in ICGC patients. (c) Survival analysis of ICGC patients was stratified by median risk score. (d) Heat map of risk groups with eight-gene signature.

To construct LASSO model with the minimum criterion, R package “glmnet” was conducted to identify prognostic genes with the strong predicting ability (Figure 2(a)). According to the optimal lambda value of the prognostic model, an eight-gene signature (TP63, STEAP3, NQO1, ELAVL1, PRKAA1, HELLS, FANCD2, and CDKN2A) was generated (Figure 2(b)). Here is the formula: Riskscores = 0.14∗Exp_TP63_ + 0.45∗Exp_STEAP3_ + 0.79∗Exp_NQO1_ + 0.14∗Exp_ELAVL1_ + (−0.11)∗Exp_PRKAA1_ + (−0.91)∗Exp_HELLS_ + (−0.16)∗Exp_FANCD2_ + (−0.41)∗Exp_CDKN2A_. Based on the median risk scores, CLL patients were divided into high- and low-risk groups. Gene expression profile of the eight genes is shown in Figure 2(c). In general, risk genes (TP63, STEAP3, NQO1, and ELAVL1) were higher expressed in high-risk group, while the protective genes (PRKAA1, HELLS, FANCD2, and CDKN2A) were higher expressed in low-risk group (Figure 2(c)). The Kaplan-Meier results showed that the high-risk group was significantly associated with a shorter OS time (Figure 2(d)).

### 3.3. Independent Prognostic Value of the Eight-Gene Signature and External Validation in a GSE Cohort

**Figure 3:**
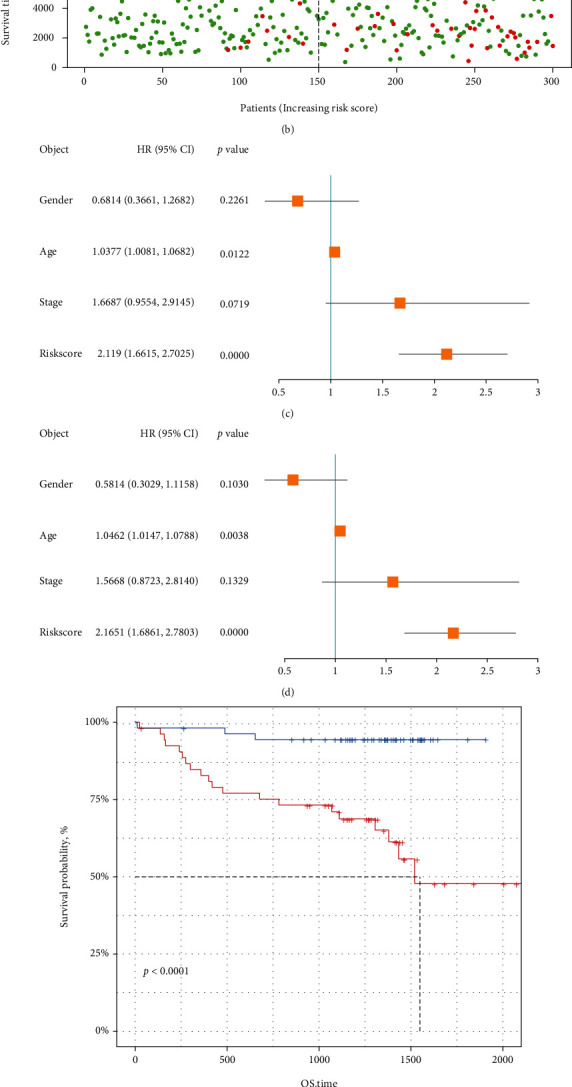
Cox regression analysis and validation of the risk signature. (a) Time-dependent ROC curves for predicting 1-, 3-, and 5-year overall survival probability in ICGC cohort. (b) Distribution of risk score, patient survival time, and status of patients. (c, d) Univariate and multivariate Cox regression analysis for the risk score and other clinicopathological features. (e) Survival analysis for the overall time of the 8 gene signature in GSE22762 cohort. (f) Time-dependent ROC curves for predicting 1-, 3-, and 5-year overall survival probability in GSE22762 cohort.

We performed ROC analysis by R package “survivalROC” to access the efficacy of eight-gene signature in predicting the clinical outcomes of CLL. ROC curve showed that the eight-gene signature had a good predictive accuracy for 1-, 3-, and 5-year OS (for 1-year, AUC =0.872; for 3-year, AUC =0.707; for 5-year, AUC =0.734) (Figure 3(a)). According to the risk scores, the CLL patients were ranked from left to right shown in the upper panel of Figure 3(b). The risk scores increased from left to right. OS distribution of each patient is shown in the lower panel of Figure 3(b), where CLL patients were ranked from left to right according to risk scores. Univariate and multivariate Cox regression analysis demonstrated that the risk score was an independent risk factor in predicting prognosis for CLL patients (Figures 3(c) and 3(d)).

Furthermore, external data “GSE22762” cohort was used to validate the predictive value of the eight-gene signature. In line with the results from the training cohort, the high-risk group had a worse OS than the low-risk group (Figure 3(e)), and the eight-gene signature showed a moderate sensitivity and specificity for 1-, 3-, and 5-year OS (for 1-year, AUCs =0.678; for 3-year, AUC =0.677; for 5-year, AUC =0.781) in the GSE cohort (Figure 3(f)).

### 3.4. Identification of Differentially Expressed Genes (DEGs) and Functional Enrichment Analysis

**Figure 4:**
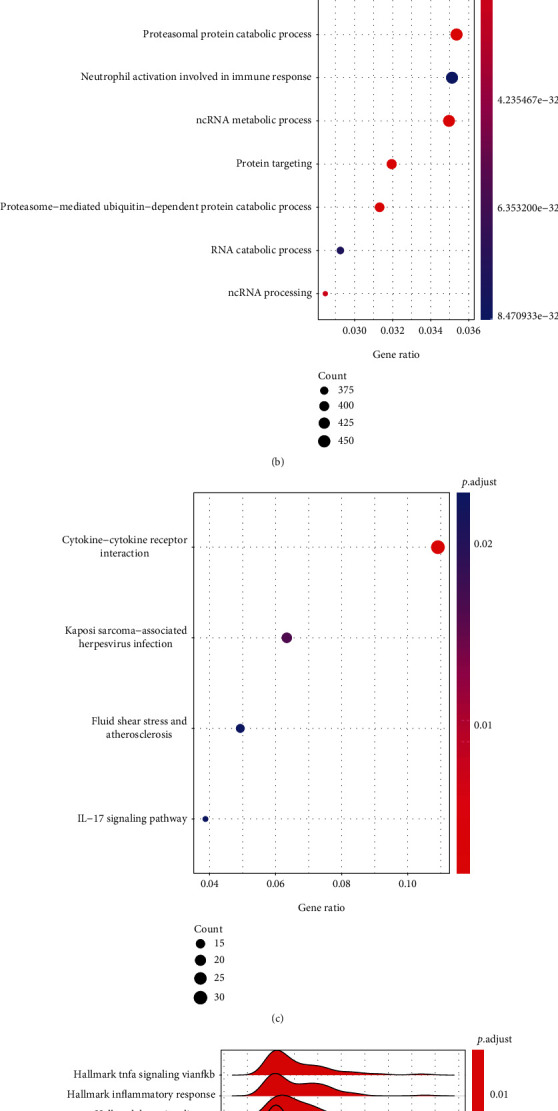
Different expression genes identification and enrichment analysis. (a) Volcanic diagram of DEGs between high-risk and low-risk groups. (b, c) The functional annotation of DEGs using GO BP terms and KEGG pathway. (d) The enrichment analysis for the 50 hallmark gene sets of tumor. At the bottom of the graph, red represented upregulated genes in high-risk patients and blue represented downregulated genes.

To better understand the biological differences between the high- and low-risk groups, R package “DESeq2” was used to analyze DEGs between the two risk groups (Figure 4(a)). Seven hundred and forty-six DEGs (|log_2_FC| >1 and FDR<0.05) were identified. Among those DEGs, 690 genes were upregulated and 56 genes were downregulated in the high-risk group compared with the low-risk group. Subsequently, “biological processes (BP)” term of Gene Ontology (GO), Kyoto Encyclopedia of Genes (KEGG) analysis, and Gene Set Enrichment Analysis (GSEA) were conducted. The BP term of GO results showed that the main biological processes involved included “autophagy” and “the process utilizing autophagic mechanisms” (Figure 4(b)). Pathway enrichment analysis with reference to KEGG focused on “cytokine−cytokine receptor interaction” and “IL−17 signaling pathway” (Figure 4(c)). The GSEA results showed that hallmark gene sets were enriched in the low-risk group, such as “TNF*α* signaling via NF-kB,” “inflammatory response,” and “IL6 JAK STAT3 signaling” (Figures 4(d)).

### 3.5. Identification of Immune Statues and Correlation Analysis

**Figure 5:**
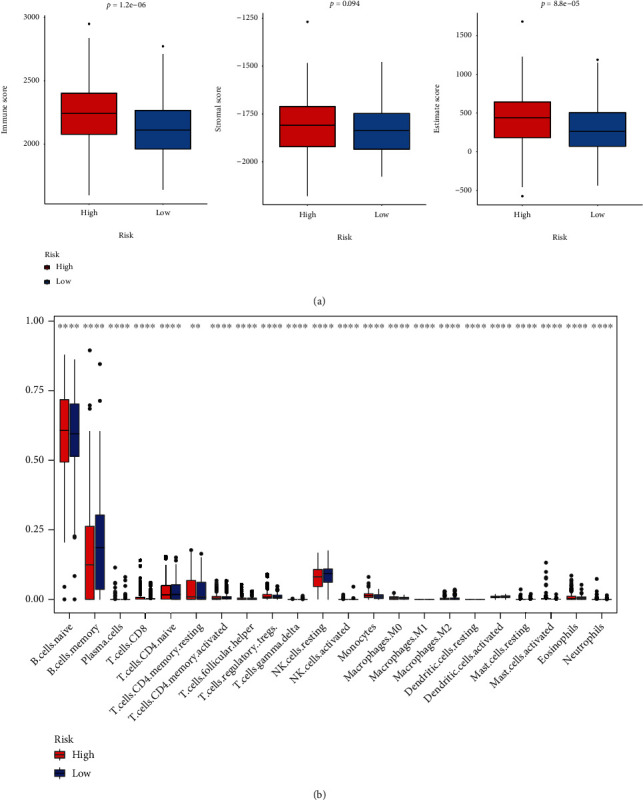
The comparison of immunity between the high- and low-risk groups. (a) The boxplot showed the difference between the high- and low-risk groups. From left to right, immune score, stromal score, and ESTIMATE score. (b) The profiles of immune infiltration between the high- and low-risk groups. ∗*p* < 0.05, ∗∗*p* < 0.01, ∗∗∗*p* < 0.001, and ∗∗∗∗*p* < 0.0001.

Recently, a few studies explored the relationship between ferroptosis and immune status [16] and immune-related gene signatures were established for predicting prognosis of CLL patients [17–20]. Moreover, the results of our enrichment analysis showed that the two risk groups had a difference in immune-related pathways. Therefore, we further investigated the TIM of the high- and low-risk groups. In terms of ESTIMATE and immune scores, the high-risk group had significantly higher values than the low-risk group (Figure 5(a)). And, there was no significant difference in the stromal scores between the two groups (Figure 5(a)). Moreover, the relative infiltration of 22 immune cells in the TIM was calculated by using CIBERSORT algorithm. The 22 immune cells were significantly differentially infiltrated between the two groups (Figure 5(b)). For example, the low-risk group had significantly more memory B cells, while the high-risk group had significantly more naive B cells.

**Figure 6:**
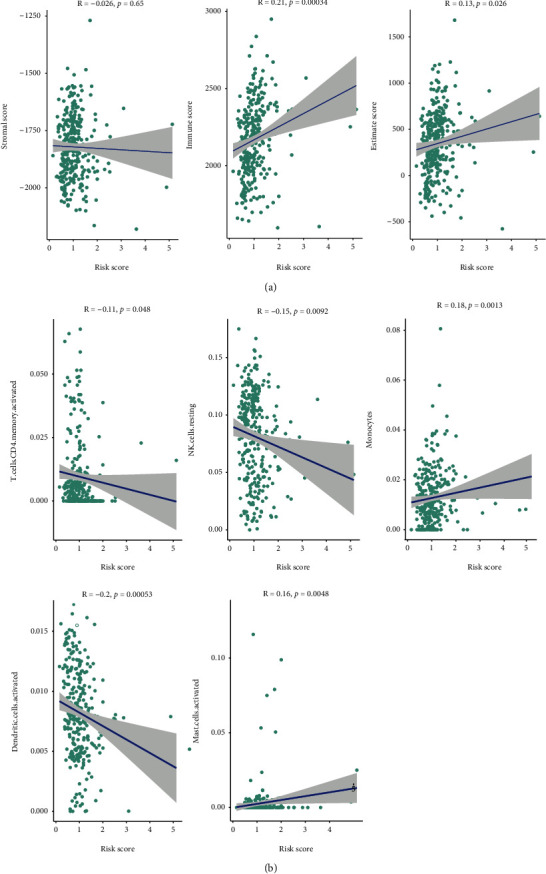
The correlation analysis of immunity with the two risk groups. (a) The correlation between the risk score and stromal score (left), immune score (middle), or ESTIMATE score (right). (b) The correlation between the risk score and immune infiltration. Five of the 22 immune cells were significantly correlation (*p* value <0.05).

The risk score had a significant correlation with the immune score and the ESTIMATE score, but was not significantly correlated with stromal scores, using Spearman's correlation analysis (Figure 6(a)). In addition, the risk score was significantly correlated with expression levels of five immune cells, such as monocytes and NK cells resting (Figure 6(b)).

### 3.6. Genomic Mutation Analysis

**Figure 7:**
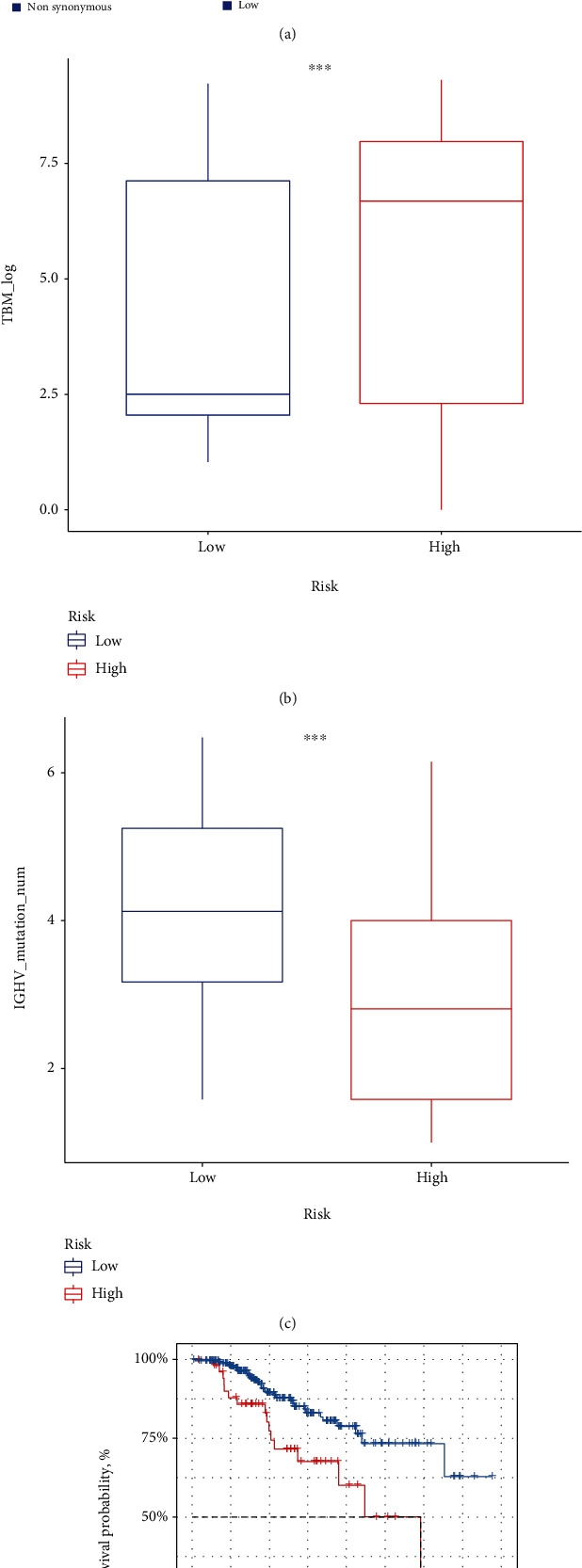
Genetic changes of the 8 signature genes between the two risk groups. (a) The waterfall plot showed the mutation profile of CLL patients in ICGC. (b) The comparison of tumor mutation burden between the high- and low-risk groups. (c) The comparison of IGHV gene mutations between the two risk groups. (d) Survival analysis of CLL patients with 8-gene signature mutation and those without. ∗*p* < 0.05, ∗∗*p* < 0.01, ∗∗∗*p* < 0.001, and ∗∗∗∗*p* < 0.0001.

In some cancers, tumor mutation burden (TMB) is an important predictive biomarker for cancer immunotherapy [21]. Differences in the somatic mutations of genes were found between the high- and low-risk groups. High-risk group had relatively higher mutation rates than the low-risk group (Figure 7(a)). Compared with the low-risk group, the high-risk group also had a higher tumor mutation burden (Figure 7(b)). Immune globulin heavy chain variable region (IGHV) gene is a classical clinical prognostic indicator for CLL [12]. Patients with low IGHV mutation rates had a worse prognosis. We found the high-risk group had a lower mutational level of IGHV gene than the low-risk group (Figure 7(c)). Moreover, mutations in the eight-gene signature were associated with a shorter OS time (Figure 7(d)).

### 3.7. Drug Resistance Analysis

**Figure 8:**
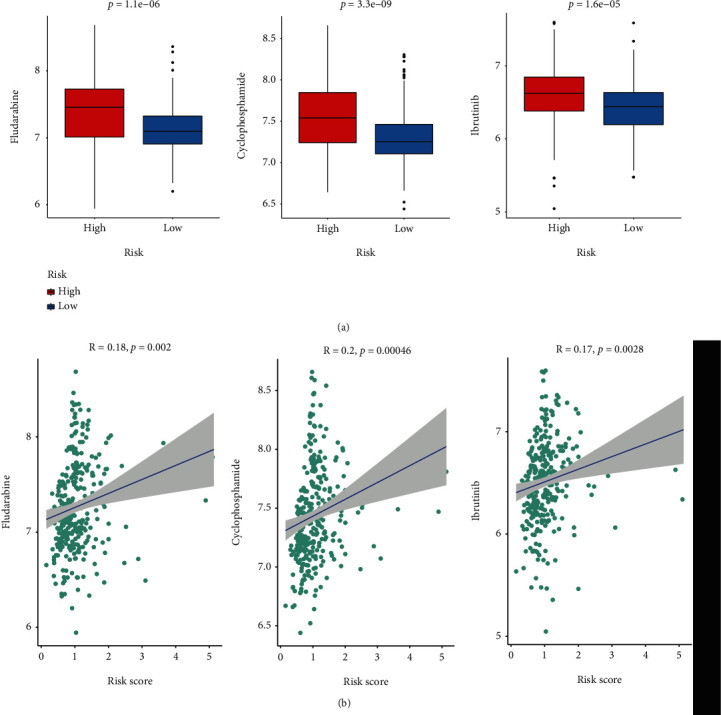
Drug sensitivity analysis. (a) Comparison of IC50 values for cyclophosphamide (left), fludarabine (middle), and ibrutinib (right) between the two risk groups. (b) The correlation analysis of the risk scores with IC50 values of the three drugs.

To investigate the potential effect of chemotherapy in the two risk groups, we conducted the R package “oncoPredict” to predict the IC_50_ value of over 200 clinical drugs in patients combined with GDSC2 drug sensitivity database. We found that the low-risk group had lower IC_50_ for three common CLL drugs compared to the high-risk group, including fludarabine, cyclophosphamide, and ibrutinib (Figure 8(a)). In addition, we found the risk scores had a significant positive correlation with IC_50_ values of the three drugs (Figure 8(b)). These results revealed that low-risk patients were more likely to benefit from fludarabine, cyclophosphamide, and ibrutinib treatment.

## 4. Discussions

CLL accounts 10% of hematologic malignancies [22] and is the most frequent subtype of leukemia in adults [8]. Ferroptosis is a new form of programmed cell death [23] and usually caused by strong membrane lipid peroxidation and oxidative stress [24]. Recently, a few studies have revealed the prognostic value of ferroptosis-related genes in malignancies [15, 25]. However, the prognosis value of ferroptosis-related genes in CLL is still unclear.

In this study, for the first time, a ferroptosis-related gene signature was established for predicting OS of CLL patients. In the training and external cohorts, CLL patients were divided into high-risk and low-risk groups by this reliable and robust signature, and the former inclined worse OS than the latter. Half of the signature genes are risk genes (TP63, STEAP3, NQO1, and ELAVL1), while the other half are protective genes (PRKAA1, HELLS, FANCD2, and CDKN2A). The eight ferroptosis-related signature genes have been suggested to associate with prognosis in cancer. TP63 contributes to maintaining redox homeostasis through glutathione biogenesis, utilization, and regeneration. TP63 is a prognostic gene in breast cancer patients [26], lung squamous cell carcinoma [27], pancreatic cancer [28], skin cutaneous melanoma [29], anaplastic lymphoma kinase-negative anaplastic large cell lymphoma [30], etc. STEAP3 is a metal reductase, encoding a transmembrane protein that functions as an iron transporter and coordinates the regulation of iron homeostasis [31]. STEAP3 can predict outcomes in clear cell renal cell carcinoma [32, 33], pancreatic adenocarcinoma [34], uveal melanoma [35] and glioblastoma [36]. NQO1 is dysregulated in many cancers and considered a target for tumor treatment and diagnosis [37]. In CLL, the increased expression of NQO1 leads to resistance to drugs that induce ROS accumulation [38]. NQO1 polymorphism is considered a risk and prognostic factor for CLL [39]. HELLS is a lymphoid-specific helicase that can lead to ferroptosis resistance by reducing the concentration of iron and lipid hyperoxide. HELLS is associated with prognosis in adrenocortical carcinoma [40], pancreatic cancer [41], soft tissue sarcoma [42], cervical cancer [43], and clear cell renal cell carcinoma [44]. PRKAA1 is a ferroptosis driver and inhibition of PRKAA/AMPK*α* diminishes ferroptosis.^35^ Genetic variations of PRKAA1 associate with prognosis for patients with colorectal cancer [45]. Recently, studies have revealed that some noncoding RNAs (ncRNAs), particularly microRNAs, long noncoding RNAs, and circular RNAs, were involved in the regulation of ferroptosis and affect cancer growth [46]. Moreover, Katsaraki et al. proposed that ncRNA plays an important role in CLL and summarizes the important discovery about their value as regulators, biomarkers, or therapeutic targets in B-CLL [47]. For example, two novel transfer RNA-derived RNA fragments (tRFs), i-tRF-Gly^GCC^ and i-tRF-Phe^GAA^, were identified as prognostic biomarkers in CLL [48, 49]. Thus, the mechanisms of ncRNA regulating the eight ferroptosis-related signature genes deserve further investigation.

To further explore the functions of DEGs between the high- and low-risk groups, we conducted GO, KEGG, and GSEA enrichment analyses. The most abundant BP term of GO was autophagy, the process utilizing autophagic mechanisms, etc. In a previous report, ferroptosis was originally defined as a programmed cell death distinct from autophagy, apoptosis, and necrosis [7]. However, recent insights suggest that ferroptosis is not a self-standing phenomenon and has close connections with other cellular events [50]. For example, ferroptosis dependents on autophagy, especially selective types of autophagy, through direct or indirect regulation of iron accumulation or lipid peroxidation [50, 51]. The top KEGG result is cytokine−cytokine receptor and the top 2 GSEA results are the hallmark gene sets of TNF*α* signaling via NF-*κ*B and inflammatory response. These results suggest significant differences in immune function between the two risk groups. In addition, the hallmark gene sets such as TNF*β* signaling and apoptosis were also enriched in the high-risk groups. They had well-documented roles in apoptosis [52–54]. Growing research suggests the interconnection of ferroptosis and apoptosis. For example, ferroptotic agents induce endoplasmic reticulum stress-mediated activation of the PERK-eIF2*α*-ATF4-CHOP pathway that is involved in the synergistic interaction between ferroptosis and apoptosis [55]. Inhibitor of apoptosis-stimulating protein of p53 inhibits ferroptosis in acute lung injury [56]. For example, TGF-*β* and TNF*α* work in concert to activate apoptosis in gastric cancer cell [57]. Ferritin as a NF-*κ*B downstream effector can inhibit TNF*α*-induced apoptosis by reducing reactive oxygen species (ROS) [58]. Moreover, deferoxamine-induced increase of the intracellular iron can activate TGF*β* and TNF*α*-dependent NF-*κ*B signaling in highly aggressive breast cancer cells [59]. Autophagy can contribute to ferroptosis through degradation of the ferritin [50]. Given the evidences above, ferroptosis, autophagy, apoptosis, and immune may together contribute to significantly different outcomes between the high- and low-risk groups.

CLL is characterized by a wide spectrum of immune dysfunctions. These immune alterations strongly impact the course and management of CLL. Immunohistochemical studies suggested that the sensitivity to ferroptosis is parallel to anticancer immunity [60]. Moreover, immune parameters have been demonstrated to associate with the prognostic relevance of patients with CLL [17–20]. In recent years, T cells inducing ferroptosis in mice bearing ovarian tumors have been reported. Immunotherapy-activated CD8+ T cells enhance ferroptosis in tumor cells, which in turn contributes to increased efficiency of the immunotherapy [16]. IFN-*γ* derived from immunotherapy-activated CD8+ T cells enhances tumor lipid oxidation and ferroptosis in human fibrosarcoma cells and melanoma cells [61]. The immune statues in the high- and low-risk groups were explored in this study. The high-risk group showed a remarked different immune profile from the low-risk group. In comparison to the low-risk group, the high-risk group exhibited a higher level of ESTIMATE score, immune score, and immune cells infiltration and thus potentially has a higher response rate to immunotherapy. In addition, TMB can predict a better response to immunotherapy in some types of cancers [62, 63]. Bufu Tang et al. use ferroptosis-related genes to stratify hepatoma patients into high- and low-risk groups, and the high-risk group has a higher TMB [21]. In our study, TMB of the high-risk group was also higher compared with the low-risk group. Recently, the value of *IGHV* gene mutation in predicting the durability of response to chemoimmunotherapy has been reported [64–66]. *IGHV* gene mutation status is one of the strongest prognostic factors currently used in clinical trials for CLL patients and associated with better clinical outcomes [12]. The OS of patients with mutated *IGHV* genes is significantly longer irrespective of the disease stage [67, 68]. The low-risk group had a higher level of *IGHV* gene mutations, which further approved our stratification of the CLL patients.

It must be acknowledged that this study has some limitations. First, the 8-gene signature needs to be validated by using larger clinical cohorts. Second, we need more experiments to explore the detailed mechanisms of the 8-gene signature in CLL. Third, the relationship between ferroptosis and immunity in CLL needs more experimental investigation.

In conclusion, by dividing CLL patients into high- and low-risk groups, a ferroptosis-related gene signature for prognostic prediction was firstly developed and validated. The biology function, immunity, and mutation status were remarkably different between the two groups. This eight-gene signature is strongly associated with OS in CLL patients and might serve as a potential prognostic biomarker for clinical use. In future work, the development of personalized treatment strategies for each patient will be an essential topic.

## Data Availability

Raw RNA sequence data that support the findings of this study are available from the CCLE-ES dataset of ICGC database (https://dcc.icgc.org/) and the GEO database of NCBI (https://www.ncbi.nlm.nih.gov/).

## Abbreviations

FRGs: Ferroptosis-related genes
CLL: Chronic lymphocytic leukemia
DEGs: Differentially expressed genes
GO: Gene ontology
BP: Biological processes
GSEA: Gene Set Enrichment Analysis
KEGG: Kyoto Encyclopedia of Genes
PCA: Principal component analysis
TMB: Tumor mutation burden
ICGC: International Cancer Genome Consortium
ROC: Receiver operating characteristic
TIM: Tumor immune microenvironment
IC_50_: Half maximal inhibitory concentration
FDR: False discovery rate
log2FC: log_2_ fold change
LASSO: The least absolute shrinkage and selection operator.

## Supplementary Materials

Supplementary Materials Table S1: 22 Immune Cell Types Description.
