# Supplementary material for: A Ferroptosis Molecular Subtype-Related Signature for Predicting Prognosis and Response to Chemotherapy in Patients with Chronic Lymphocytic Leukemia
Source: Biomed Res Int. 2022 Jul 6;2022:5646275. doi: 10.1155/2022/5646275 (PMC9279058; doi:10.1155/2022/5646275)
Supplement: Supplementary Materials — Table S1: 22 Immune Cell Types Description. [file 5646275.f1.docx]

| Supplementary Table S1: 22 Immune Cell Types Description | |
| --- | --- |
| LM22 Cells | 22 Cell Type Description |
| B cells | B cells naive |
|  | B cells memory |
| PCs | Plasma cells |
| CD8 T | T cells CD8 |
| CD4 T cells | T cells CD4 naive |
|  | T cells CD4 memory resting |
|  | T cells CD4 memory activated |
|  | T cells follicular helper |
|  | T cells regulatory (Tregs) |
| Gamma delta T cells | T cells gamma delta |
| NK cells | NK cells resting |
|  | NK cells activated |
| Monocytes and Macrophages | Monocytes |
|  | Macrophages M0 |
|  | Macrophages M1 |
|  | Macrophages M2 |
| Dendritic cells | Dendritic cells resting |
|  | Dendritic cells activated |
| Mast cells | Mast cells resting |
|  | Mast cells activated |
| Eos | Eosinophils |
| PMNs | Neutrophils |
